# Secondary Prevention of Suicidal Behaviours Through a 12-month Telephone-Based Programme: Treatment Adherence and Clinical Outcomes

**DOI:** 10.1192/j.eurpsy.2026.12079

**Published:** 2026-07-31

**Authors:** L. Comendador Vázquez, A. I. Cebrià, A. Sanz, D. J. Palao, M. Diaz-Marsá, M. Elices, I. Grande, M. P. López-Peña, Á. Palao-Tarrero, A. Pedrola-Pons, M. Ridao, M. Ruiz-Veguilla, P. Saiz, A. de la Torre-Luque, V. Perez-Sola

**Affiliations:** 1 Department of Psychiatry and Forensic Medicine, Faculty of Medicine, Universitat Autònoma de Barcelona, Cerdanyola del Vallès; 2 Department of Mental Health, Parc Taulí University Hospital, Institut d’Investigació i Innovació Parc Taulí (I3PT-CERCA), Sabadell; 3 Centro de Investigación Biomédica en Red de Salud Mental (CIBERSAM), Instituto de Salud Carlos III, Madrid; 4 Department of Clinical and Health Psychology, Faculty of Psychology; 5 Department of Basic, Developmental, and Educational Psychology, Faculty of Psychology, Universitat Autònoma de Barcelona, Cerdanyola del Vallès; 6 Hospital Clinico San Carlos; 7 Department of Legal Medicine, Psychiatry and Pathology. School of Medicine, Universidad Complutense de Madrid, Madrid; 8 Neuroscience Research Program, Hospital del Mar Research Institute, Hospital del Mar; 9 Departament de Medicina, Facultat de Medicina i Ciències de la Salut, Universitat de Barcelona; 10 Bipolar and Depressive Disorders Unit, Hospital Clinic de Barcelona; 11 Institut d’Investigacions Biomèdiques August Pi i Sunyer (IDIBAPS); 12 Institute of Neurosciences (UBNeuro), Barcelona; 13 BIORABA, Department of Psychiatry, Hospital Universitario de Alava, UPV/EHU; 14 UPV/EHU, Vitoria; 15 Hospital La Paz Institute for Health Research (IdiPAZ); 16 Department of Psychiatry, Universidad Autónoma de Madrid; 17 Department of Psychiatry, Clinical Psychology and Mental Health, La Paz University Hospital; 18 Department of Legal Medicine, Psychiatry and Pathology. Faculty of Medicine, Complutense University of Madrid, Madrid; 19 Hospital Virgen del Rocio, IBIS; 20 Universidad de Sevilla, Seville; 21 Department of Psychiatry, Universidad de Oviedo; 22 Instituto de Investigación Sanitaria del Principado de Asturias (ISPA); 23 Instituto Universitario de Neurociencias del Principado de Asturias (INEUROPA); 24 Servicio de Salud del Principado de Asturias (SESPA), Oviedo; 25 Mental Health Institute, Hospital del Mar; 26 Department of Experimental and Health Sciences, Pompeu Fabra University, Barcelona, Spain

## Abstract

**Introduction:**

Suicidal behaviour is a major public health concern. After hospital discharge, the probability of recurrence is particularly high, underlining the importance of structured follow-up care. Telephone-based interventions have emerged as an accessible and cost-effective strategy to provide continuous support to individuals at risk. These programmes have shown potential to reduce reattempts and improve engagement with treatment, although adherence often remains a challenge.

**Objectives:**

The present study examines the effects of a 12-month telephone follow-up intervention as a secondary prevention strategy and explores the clinical and sociodemographic factors associated with adherence in adults with a previous suicide attempt.

**Methods:**

A coordinated, multi-centre randomised controlled clinical trial was conducted to test telephone-managed secondary prevention programmes to prevent suicide compared to treatment as usual (TAU), as a part of a prospective cohort of individuals presenting a suicide attempt (SURVIVE project). The primary outcome was the subsequent suicide attempts. The secondary variables were adherence to the telephone intervention programme; characteristics of the suicide attempt; sociodemographic variables; impulsivity; adherence to pharmacological treatment; and acquired capacity for suicide, among others. A total of 600 participants were recruited, *n* = 300 in the telephone management intervention group and *n* = 300 in the control group (TAU). The protocol has been approved by the ethics committees (EC) of each participating centre.

**Results:**

The results of the study provide relevant information on adherence to a suicide prevention programme based on telephone follow-up and its effects on the recurrence of suicidal behaviour. In general, it was observed that adherence to the telephone intervention is moderated by sociodemographic and clinical variables.

**Conclusions:**

Understanding which factors predict adherence to telehealth interventions following a suicide attempt is essential to assess both their feasibility, long-term applicability, and effectiveness. Exploring clinical and psychosocial profiles linked to treatment engagement among individuals discharged from the emergency department after a suicide attempt could provide valuable insights and foster improvements in clinical practice.

**Disclosure of Interest:**

L. Comendador Vázquez Grant / Research support from: This study was supported by the Instituto de Salud Carlos III-FIS research grants (PI19/00236, PI19/00569, PI19/00685, PI19/00941, PI19/00954, PI19/01027, PI19/01256, PI19/01484, PI20/00229 and PI23/00147, PI23/01483, PI23/00822, PI23/00614, PI23/00085, PI23/00707, PI23/01469, PI23/01277, PI23/01367, PI23/01066), co-funded by the European Regional Development Fund (ERDF) “A Way to Build Europe”., A. I. Cebrià: None Declared, A. Sanz: None Declared, D. J. Palao Consultant of: D.P. has received grants and served as a consultant or advisor for Rovi, Angelini, Janssen, Lundbeck and Servier., M. Diaz-Marsá: None Declared, M. Elices: None Declared, I. Grande Consultant of: I.G. has received grants and has served as a consultant, advisor or CME speaker for the following entities: ADAMED, Angelini, Casen Recordati, Esteve, Ferrer, Gedeon Richter, Janssen Cilag, Lundbeck, Lundbeck-Otsuka, Luye, SEI Healthcare, Viatris outside the submitted work. She also receives royalties from Oxford University Press, Elsevier, Editorial Médica Panamericana., M. P. López-Peña Grant / Research support from: P.L.P. in the last 5 years has received collaborations in the form of registration fees for courses and congresses from the following industries: Janssen-Cilag, Lundbeck, Otsuka, Pfizer, Angelini, Novartis, Rovi, Takeda., Á. Palao-Tarrero: None Declared, A. Pedrola-Pons: None Declared, M. Ridao: None Declared, M. Ruiz-Veguilla: None Declared, P. Saiz Consultant of: P.S. has been a consultant to and/or has received honoraria or grants from Adamed, Alter Medica, Angelini Pharma, “laCaixa Foundation”, CIBERSAM, Ethypharm Digital Therapy, European Commission, Government of the Principality of Asturias, Instituto de Salud Carlos III, Johnson & Johnson, Lundbeck, Otsuka, Pfizer, Plan Nacional Sobre Drogas, Rovi, Servier, and Viatris España outside the submitted work., A. de la Torre-Luque: None Declared, V. Perez-Sola Consultant of: V.P. has been a consultant to or has received honoraria or grants from AB-Biotics, AstraZeneca, Bristol-Myers-Squibb, CIBERSAM, FIS- ISCiii, Janssen Cilag, Lundbeck, Otsuka, Servier and Medtronic.

